# Assessment of Secular Trends and Health Risk in Pediatric Cardiorespiratory Fitness From the Republic of Slovenia

**DOI:** 10.3389/fphys.2021.644781

**Published:** 2021-03-08

**Authors:** Shawnda A. Morrison, Vedrana Sember, Bojan Leskošek, Marjeta Kovač, Gregor Jurak, Gregor Starc

**Affiliations:** Faculty of Sport, University of Ljubljana, Ljubljana, Slovenia

**Keywords:** health risk, generation, shuttle run, temporal trends, population health, youth

## Abstract

**Objectives:**

Determine the temporal trends in cardiorespiratory fitness (CRF) and health risk of Slovenian schoolchildren across a 20-year span, assessed via 20-m shuttle run (20mSRT), including defining centile ranges and possible health risk(s) for each generation.

**Methods:**

Nationally representative data from 9,426 healthy schoolchildren (6–14 years old) were used to determine changes in CRF across three generations, in 1993 (*n* = 3,174), 2003 (*n* = 3,457) and 2013 (*n* = 2,795) from a multistage, stratified, decennial study.

**Results:**

20mSRT performance declined ∼2.8% from 1993 to 2003, independent of age or sex of the child. This trend was reversed in 2013, increasing by ∼8.2% across all age groups, for both girls and boys, for a net increase of 5.4%. The magnitude of improvement was similar for both sexes. Moreover, girls in the 2013 generation (for ages 10–13 year) completed more stages than their 2003 male counterparts. Across all generations, children achieved CRF values corresponding to low cardiovascular risk for future health outcomes. Centile values ranged from “low” to “very high” depending on age, sex, and generation of the sample.

**Conclusion:**

Negative trends in CRF from Slovenian schoolchildren were reversed by 2013, indicating that Slovenia should continue implementing progressive national physical fitness strategies introduced between sampling periods (i.e., 2003–2013). Additionally, due to the universal nature of Slovenian schoolchildren achieving “healthy cut-off values” for 20mSRT (generation-inclusive), it is suggested that more specific cut-off criteria are developed, especially for younger children, and girls, so that future CRF results can be more accurately applied for both clinical and pedagogical users.

## Introduction

The 20-m shuttle run test (20mSRT), is an indirect measure of cardiorespiratory fitness (CRF) and one of the most widely used field measures of CRF, especially amongst children and youth (Lang et al., 2018b). Briefly, the 20mSRT involves continuous running back and forth between lines 20-m apart, in time to audio signals. It consists of multiple stages which last ∼1 min and each stage comprises of a given number of laps. With every stage progression, the required running speed increases until volitional fatigue or the participant no longer completes the distance in-line with the audio signal on two consecutive occasions. There are multiple variations on the original Léger protocol (Léger et al., 1984, 1988), although the most widely-reported one remains the original. Worldwide secular trends in 20mSRT performance, and issues surrounding protocol standardization, have been reviewed in detail elsewhere (Tomkinson et al., 2003, 2019b; Olds et al., 2006). More recent literature has emphasized the importance of what the 20mSRT test can, and cannot, measure effectively (reviewed in Tomkinson et al., 2019a). Indeed, at its core the 20mSRT is not a direct measure of peak oxygen consumption (V̇O_2peak_) and as such, is not able to comment on the exact amount of oxygen a moving body can take in, transport and use. Some authors have criticized the test’s ability to adequately estimate CRF, instead suggesting it better estimates body fatness (Armstrong and Welsman, 2019; Welsman and Armstrong, 2019), although this argument does not appear to consider that the 20mSRT can be an appropriate predictor of future health in its own right (García-Hermoso et al., 2020), and a suitable marker of current health status (Ramírez-Vélez et al., 2017; Lang et al., 2018a), independent of adiposity (Machado-Rodrigues et al., 2014). Thus, the 20mSRT remains a useful and widely accessible test for population health research, especially when it is conducted in a standardized way, and provided the resultant measurements are interpreted within the scope of the tool.

A systematic review and meta-analysis determined that children and youth who fall below certain CRF cut-off points are at increased risk of cardiovascular disease (Ruiz et al., 2016). The comprehensive study included 9,280 children from 14 countries, aged 8 to 19 year, with 49% girls, and report V̇O_2peak_ cut-off point ≤35 and ≤42 mL⋅kg^–1^⋅min^–1^ for girls and boys, respectively, who are deemed to be at greater health risk (Ruiz et al., 2016). The authors determined a 20mSRT running speed of 9.0 km/h as an optimal cut-off point for detecting abdominal obesity in both sexes, across all ages (based on the original Léger protocol). The global, age-standardized prevalence of child obesity continues to increase from 0.7 to 0.9% (mean range) in 1975 to 4.8–9.1% (mean range) in 2016 (NCD Risk Factor Collaboration [NCD-RisC], 2017), although Slovenia has largely reversed this trend in both sexes from 2009 to 2020 (Sorić et al., 2020) (notably before the COVID-19 crisis). It remains whether positive trends would also be observed for objective measures of physical fitness, including cardiorespiratory health.

Thus, the purpose of this study was two-fold, (1) to identify any directional trends in CRF for Slovenian schoolchildren and (2) to determine how Slovenian schoolchildren compare to international health standards. Based on worldwide CRF data trends, it was hypothesized that CRF would decrease from 1993 to 2013 consistent across age, in both boys and girls.

## Materials and Methods

Data were collected within the ACDSi study, approved by the Slovenian National Medical Ethics Committee (ID: 138/05/13), following the Declaration of Helsinki. All data are anonymous.

### Patient and Public Involvement

Written, informed consent was obtained from parents or legal guardians of all children before voluntary participation; children could withdraw from the study, in whole or in part, anytime they wished. Schools, parents and children are kept informed of study progress through various communication methods (e.g., web pages, regular presentations at schools, etc.) where they could provide feedback on their experiences.

### Study Population

The ACDSi study investigates children’s biological, psychological and social development, described in detail elsewhere (Jurak et al., 2013). It is a cross-sectional decennial study that includes 11 primary schools. The ACDSi has been repeated four times since 1970/71; first in 1983 (Šturm and Strel, 1984) and every decade thereafter [1993 (Strel et al., 1996), 2003 (Kovač et al., 2004) and 2013 (Jurak et al., 2013)]. A national, representative sample was selected using a multi-stage, stratified design. Ten research sites were selected according to four Slovenian settlement types (village, rural town, industrial town, and city) and regions. One primary school was selected from each project site, except in the capital region where an additional school was added in 1993, in order to meet the population representation criteria at that time. Thereafter, each research cycle includes ∼3,500 children aged ∼7 to 14 years (1993 *n* = 3,488, 2003 *n* = 4,095, 2013 *n* = 3,478), representing ∼2% of Slovenia’s entire population. Specific sample sizes of children and adolescents for the current study are provided in the results section under Table 1. Note that the vast majority (>99%) of Slovenian schoolchildren attend the public-school system in the country. Private primary schools are rare and that do exist are mandated to follow the same criteria in terms of academic curricula.

**TABLE 1:** Descriptive characteristics stratified by age, sex, and generation.

**Variable**		**Boys**			**Girls**		
	**Age**	**1993**	**2003**	**2013**	**1993**	**2003**	**2013**
Sample size (N)	7 8 9 10 11 12 13 14 Total	167 205 233 208 199 204 214 215 1645	235 211 232 221 242 228 236 232 1837	203 204 195 174 168 169 141 200 1454	197 236 212 232 201 209 224 198 1709	229 224 224 244 226 213 204 210 1774	236 211 180 191 186 153 142 156 1455
Height (cm)	7 8 9 10 11 12 13 14 Mean	124.3 ± 5.5 129.9 ± 5.6 135.8 ± 5.6 141.3 ± 5.8 145.8 ± 6.5 151.9 ± 6.5 158.2 ± 8.4 166.7 ± 9.2 144.4 ± 4.0	123.8 ± 5.6 130.0 ± 6.1 134.3 ± 6.0 140.3 ± 6.7 145.7 ± 7.1 150.9 ± 7.3 158.9 ± 8.7 165.1 ± 8.3 143.8 ± 15.2^a^	125.0 ± 5.4 131.2 ± 5.4 136.4 ± 5.6 142.6 ± 7.5 148.2 ± 7.5 153.4 ± 7.2 160.0 ± 8.0 167.3 ± 7.9 145.0 ± 15.4	124.6 ± 6.0 129.5 ± 5.9 135.9 ± 6.2 140.7 ± 7.0 147.7 ± 7.5 153.8 ± 6.8 158.4 ± 6.9 162.7 ± 6.7 144.0 ± 14.4	123.6 ± 5.9 128.9 ± 5.7 135.5 ± 6.0 140.1 ± 7.0 147.2 ± 7.1 153.8 ± 7.0 158.3 ± 7.5 161.1 ± 5.9 143.0 ± 14.3^a^	125.1 ± 5.3 130.2 ± 5.6 136.8 ± 6.2 142.4 ± 6.2 149.5 ± 7.2 156.0 ± 6.7 159.8 ± 6.5 162.9 ± 5.6 143.6 ± 14.6
Mass (kg)	7 8 9 10 11 12 13 14 Mean	25.2 ± 4.7 28.0 ± 5.4 31.9 ± 6.5 35.1 ± 6.8 38.6 ± 7.9 43.4 ± 9.4 48.5 ± 10.6 56.6 ± 11.9 38.5 ± 12.9^b,c^	25.5 ± 4.7 29.6 ± 6.7 32.6 ± 6.9 35.8 ± 8.1 39.5 ± 8.4 43.5 ± 9.3 50.6 ± 11.1 56.1 ± 11.4 39.3 ± 13.1^a,c^	25.7 ± 4.7 29.4 ± 5.6 33.4 ± 6.9 38.2 ± 8.6 42.8 ± 10.7 46.4 ± 10.9 52.9 ± 13.3 59.3 ± 12.0 40.6 ± 14.5^a,b^	24.9 ± 5.2 27.5 ± 5.5 31.5 ± 6.4 34.5 ± 7.4 39.3 ± 8.7 44.5 ± 9.0 49.5 ± 9.6 53.8 ± 9.2 38.1 ± 12.4^b,c^	25.6 ± 5.5 28.6 ± 5.9 33.0 ± 6.7 36.0 ± 8.1 40.8 ± 8.4 46.8 ± 10.2 51.7 ± 11.1 53.9 ± 8.5 39.1 ± 12.7^a,c^	25.6 ± 5.5 29.3 ± 6.1 33.1 ± 6.7 37.8 ± 9.0 43.8 ± 9.5 49.2 ± 12.2 52.7 ± 10.5 54.6 ± 9.1 39.4 ± 13.4^a,b^
BMI (kg/m^2^)	7 8 9 10 11 12 13 14 Mean	16.2 ± 2.2 16.5 ± 2.3 17.2 ± 2.6 17.4 ± 2.6 18.1 ± 3.0 18.7 ± 3.2 19.2 ± 2.9 20.2 ± 3.2 18.0 ± 3.1^b,c^	16.5 ± 2.0 17.4 ± 2.9 17.9 ± 2.9 18.0 ± 2.9 18.5 ± 2.9 19.0 ± 3.1 19.9 ± 3.2 20.4 ± 3.2 18.5 ± 3.1^a^	16.4 ± 2.3 17.0 ± 2.5 17.8 ± 2.8 18.7 ± 3.2 19.3 ± 3.7 19.6 ± 3.6 20.5 ± 3.9 21.1 ± 3.4 18.7 ± 3.6^a^	15.9 ± 2.5 16.3 ± 2.6 16.9 ± 2.6 17.3 ± 2.8 17.9 ± 2.9 18.7 ± 2.9 19.6 ± 3.2 20.3 ± 2.9 17.9 ± 3.2^b,c^	16.6 ± 2.5 17.1 ± 2.6 17.9 ± 2.8 18.2 ± 3.0 18.7 ± 2.8 19.6 ± 3.2 20.5 ± 3.5 20.7 ± 2.8 18.6 ± 3.2^a^	16.3 ± 2.6 17.2 ± 2.8 17.6 ± 2.7 18.5 ± 3.5 19.5 ± 3.4 20.0 ± 4.1 20.6 ± 3.4 20.5 ± 3.0 18.6 ± 3.5^a^
BMI (z-score)	7 8 9 10 11 12 13 14 Mean	−0.75 ± 0.61 −0.60 ± 0.67 −0.42 ± 0.79 −0.33 ± 0.74 −0.16 ± 0.97 −0.06 ± 0.82 0.19 ± 0.88 0.40 ± 0.92 −0.22 ± 0.80^b,c^	−0.61 ± 0.64 −0.34 ± 0.82 −0.29 ± 0.79 −0.20 ± 0.86 −0.09 ± 0.80 0.09 ± 0.92 0.32 ± 0.85 0.48 ± 0.89 −0.08 ± 0.82^a^	−0.70 ± 0.61 −0.42 ± 0.79 −0.26 ± 0.83 0.01 ± 0.92 0.14 ± 1.04 0.31 ± 1.05 0.51 ± 1.03 0.64 ± 0.94 0.03 ± 0.91^a^	−0.80 ± 0.72 −0.61 ± 0.76 −0.60 ± 0.73 −0.36 ± 0.78 −0.15 ± 0.88 −0.02 ± 0.84 0.25 ± 0.85 0.48 ± 0.73 −0.23 ± 0.79^b,c^	−0.57 ± 0.76 −0.49 ± 0.71 −0.23 ± 0.84 −0.21 ± 0.80 0.06 ± 0.85 0.19 ± 0.90 0.54 ± 0.90 0.52 ± 0.82 −0.02 ± 0.82^a^	−0.67 ± 0.78 −0.40 ± 0.75 −0.33 ± 0.87 0.02 ± 0.99 0.18 ± 1.06 0.41 ± 1.09 0.39 ± 0.83 0.55 ± 0.87 0.02 ± 0.91^a^
Triceps skinfold (mm)	7 8 9 10 11 12 13 14 Mean	9.2 ± 3.9^e^ 9.5 ± 4.0^g^ 11.0 ± 5.4 11.0 ± 5.0 11.2 ± 5.3 11.1 ± 5.6 10.7 ± 5.8 10.1 ± 5.1 10.5 ± 5.1^b,c^	10.3 ± 3.9 ^e^ 11.5 ± 4.6^g^ 11.5 ± 4.4 12.0 ± 4.6 12.7 ± 4.9 12.1 ± 5.3 11.7 ± 5.5 11.5 ± 5.0 11.6 ± 4.8^a,c^	10.3 ± 3.8^e^ 11.8 ± 4.8^g^ 12.2 ± 5.0 13.1 ± 5.4 14.2 ± 6.6 13.6 ± 5.4 12.8 ± 6.1 12.0 ± 5.4 12.4 ± 5.4^a,b^	10.9 ± 4.1^e^ 12.0 ± 4.7^g^ 12.1 ± 4.8 12.7 ± 5.2 12.3 ± 4.5 12.5 ± 5.1 13.4 ± 4.7 14.4 ± 4.6 12.4 ± 4.8^b,c,d^	12.0 ± 4.2^e^ 12.9 ± 4.4^g^ 14.0 ± 4.8 13.3 ± 4.6 13.8 ± 4.7 13.6 ± 4.3 14.5 ± 4.7 14.3 ± 4.4 13.5 ± 4.6^a,c,d^	11.8 ± 4.4^e^ 13.4 ± 4.8 ^g^ 14.3 ± 5.2 15.8 ± 6.4 15.1 ± 5.9 15.9 ± 6.0 15.9 ± 5.3 16.4 ± 5.6 14.6 ± 5.6^a,b,d^
*Screen time (min⋅d)	7 8 9 10 Mean 11 12 13 14 Mean	– – – – – – – – – –	– – – – – – – – – –	107.7 ± 64.8 121.6 ± 85.0 130.4 ± 83.5 148.7 ± 102.6^f^ 126.0 ± 85.3 289.9 ± 187.9 321.7 ± 190.3 334.8 ± 162.2 347.8 ± 191.8 330.0 ± 184.1	– – – – – – – – – –	– – – – – – – – – –	97.5 ± 82.2 106.0 ± 76.2 112.2 ± 82.6 130.1 ± 91.5^f^ 110.5 ± 83.4 257.7 ± 179.8^d^ 250.0 ± 141.5 255.0 ± 147.3 273.4 ± 122.9 258.8 ± 145.2
MVPA (min⋅d)	7 8 9 10 11 12 13 14 Mean	– – – – – – – – –	– – – – – – – – –	– – – – 133.1 ± 95.2 126.1 ± 78.9 127.7 ± 78.6 127.7 ± 70.1 129.8 ± 76.4	– – – – – – – – –	– – – – – – – – –	– – – – 118.1 ± 71.8 102.9 ± 72.4^d^ 95.4 ± 62.6^d^ 92.2 ± 63.3^d^ 100.0 ± 67.3
Sleep (h)	7 8 9 10 11 12 13 14 Mean	– – – – – – – – –	– – – – – – – – –	– – – – 9.8 ± 1.3^e^ 9.4 ± 1.2^g^ 9.0 ± 1.2 8.9 ± 1.1 9.3 ± 1.2	– – – – – – – – –	– – – – – – – – –	– – – – 9.9 ± 1.1^g^ 9.7 ± 1.0^g^ 9.3 ± 0.9 9.2 ± 1.0 9.6 ± 1.1

*BMI, body mass index; MVPA, moderate-to-vigorous physical activity, objectively measured.*

*(^*a*^) Significantly different from 1993 generation, within-sex,*

*(^*b*^) Significantly different from 2003 generation, within-sex,*

*(^*c*^) Significantly different from 2013 generation, within-sex,*

*(^*d*^) Significantly different from boys of same generation,*

*(^*e*^) Significantly different from all other ages, within-sex for that generation.*

*(^*f*^) Significantly different than age 7-year-olds, within-sex for that generation*

(^*g*^) Different than age 14-year-olds, within-sex for that generation *p* < 0.01. Data are means ± standard deviations. (*) Denotes two methods of data collection which were analyzed separately; children aged 7–10 screen time was determined using CLASS questionnaire (i.e., parental self-reported data for the very young children), whereas children aged 11–14 used SHAPES questionnaire, where they answered on their own behalf.

### Data Collection

Despite unavoidable, slight variations in data collection methods between cycles, the full dataset consists of ≥25 anthropometric variables, ≥14 motor and aerobic fitness variables (covering most tests from Eurofit and other test batteries from the SLOfit longitudinal database), described elsewhere (Jurak et al., 2020a). Data collection for each cycle was performed by a team of researchers well-familiarized with all test protocols. Fitness testing took place indoors (room temperature ranged between 20 and 24°C), between 8:00 and 14:00 lasting 2 or 3 days for each school involved. All data collection took place in the academic fall term, from September-October.

### Measurements

#### Anthropometry

Height was measured to the nearest millimeter using a GPM 101 anthropometer (SiberHegner, Zurich, Switzerland); body mass was measured to the nearest 100 grams using a portable Tanita BWB-800P electronic scale (Arlington Heights, IL, United States). The electronic scale was calibrated each time it was moved. Skinfolds were measured to the nearest millimeter with Harpenden fat calipers (John Bull British Indicators Ltd., London, United Kingdom). Three measurements were taken at each measuring site on the right side of the body, with the mean value of the two closest measurements used for analysis.

#### Assessment of Cardiorespiratory Fitness

We used the Léger’s original 1-min 20-m shuttle run protocol (Léger et al., 1988) which starts at a speed of 8.5 km/h and increases by 0.5 km/h every minute thereafter. The test was conducted indoors, in a gymnasium, with the children barefoot. Heart rate was monitored with Polar F11 heart-rate monitors (Polar Electro, Kempele, Finland) for the 2013 generation. Resting heart rate was documented at 1 min, 2.5 min, at volitional fatigue and 5 min after test completion. Before commencing, an investigator explained the execution of the test to the children. Testing began with brief, light warm-up tasks (up to 10 min). During the test, children were not additionally verbally encouraged. The test was terminated when the participant failed to reach the end line in time with the audio signal on two consecutive occasions, or upon volitional fatigue, whichever occurred first. CRF was estimated using the following:

$$\dot{\text{V}}\,{\text{O}}_{2\,p\,e\,a\,k}=31.025+3.238\cdot \mathrm{X1}-3.248\cdot \mathrm{X2}+0.1536\cdot \mathrm{X1}\cdot \mathrm{X2}$$

where X1 is the running speed at the last completed stage (km/hour) and X2 is the age at last birthday (Léger et al., 1988).

#### Assessment of Physical Activity, Screen Time and Sleep

Physical activity (PA) was assessed with The School Health Action, and Evaluation System (SHAPES) (Leatherdale et al., 2009) for children aged 11–14 years, and the Children’s Leisure Activities Study Survey (CLASS) printed version of parental proxy self-report questionnaire (Telford et al., 2004) for younger children aged 6–11 years. Both questionnaires were back-translated from English to Slovenian by four native Slovenian speakers, following World Health Organization recommendations for translation and adaptation of instruments (World Health Organization [WHO], 2009). Both questionnaires have acceptable reliability. Validity is more desirable in SHAPES (coeff range: 0.25–0.44) versus CLASS (coeff range: 0.02–0.82) (Adamo et al., 2009), although CLASS remains one of the best proxy instruments for younger children (Chinapaw et al., 2010). Moderate-to-vigorous PA (MVPA) was calculated based on daily self-reported MVPA minutes. Total screen time was a summation of time spent watching television, watching videos on computer or DVD, using cell phone, playing videogames, and browsing the internet. The Pediatric Daytime Sleepiness Scale was used to determine total sleep time in children aged 11–14 during weekdays and weekends (Drake et al., 2003). Variables (MVPA, screen time, sleep time) are calculated separately for weekdays/weekends and summed for a total minutes per week value.

#### Statistical Analysis

A univariate ANOVA was used to describe changes in CRF between three generations, incorporating three between-subject factors (sex: 2 levels, male and female; generation: 3 levels, 1993, 2003, and 2013; age: 8 levels, ages 7–14, trunk age categories, e.g., 7.00–7.99 years, inclusive). Descriptive statistics on all relevant dependent measures are presented as means and standard deviations, and 95% confidence intervals (CI) when appropriate. *Post hoc* analyses for multiple comparisons (Sidak, listwise) were conducted when significant main effects were found at *p* < 0.01 level of significance to account for multiple comparisons. Delta change scores were calculated between the 2003 and 2013 generations for CRF and peak heart rate (within age and between sex) to determine whether changes in CRF were associated with alterations in cardiovascular strain at end stage completed. Data were initially checked for outlier analyses by examining plots visually and removing any outliers which were ± 3.00 outside the expected range of Standard Error of Estimate in each age category, and for both sexes. Total sample size was decreased 2.4% after removing the outlier data. All numbers reported in the current manuscript reflect data after initial outlier checks. Data were analyzed using IBM SPSS v.26 (Chicago, IL, United States).

## Results

### Descriptive Characteristics

Children differed (slightly) in height from 1993 to 2003 (*p* < 0.001; 95% CI: 0.4 to 1.2 cm), whilst mean mass was different for each generation (*p* < 0.001; 95% CI 1993: 38.0−38.6; 2003: 39.1−39.6; 2013: 40.6−41.2 kg, Table 1). There were no differences in BMI between girls and boys (*p* = 0.577; 95% CI: −0.15−0.083 kg⋅m^–2^) *per se*, although BMI did differ by generation (*p* < 0.001). Triceps thickness demonstrated a significant two-way interaction (age by sex, and generation by age, *p* < 0.001, Table 1).

### 20mSRT Performance Outcomes

There were significant main effects observed for sex, generation, and age (each comparison at *p* < 0.001), including significant two-way interactions for both sex by age and generation by age (*p* < 0.001). *Post hoc* comparisons indicate that each generation was unique. Boys were able to complete ∼1 higher end stage than girls (boys: 4.8, 95% CI: from 4.76 to 4.85; girls 3.9, 95% CI from 3.85 to 3.95, *p* < 0.001, Table 2). With 1993 serving as base year, end stages completed decreased by ∼half a stage (95% CI: −0.61−−0.42) in 2003, before catapulting in 2013 to children completing ∼1 full stage more than their 1993 counterparts (95% CI: 0.89−1.10, *p* < 0.001). These results translate into boys registering marginally higher V̇O_2peak_ than girls (95% CI: 2.1−2.4 mL⋅kg^–1^⋅min^–1^, *p* < 0.001) when collapsed across age and generation, and, like with end stage, each generation was unique from the other, with greatest differences occurring from 2003 to 2013 (95% CI: 3.5−4.0 mL⋅kg^–1^⋅min^–1^, *p* < 0.001). However, because there was no significant two-way interaction of sex by generation (*p* = 0.041), it can be stated that the greatest trend in this secular data is one which occurs between generations, not between the sexes. It is notable that the slope of change in V̇O_2peak_ is steepest in the 2003 girls’ generation (−0.86 mL⋅kg^–1^⋅min^–1^ per year), which is, at minimum, three times the rate of decrease observed in boys from any generation (range: −0.04−−0.26 mL⋅kg^–1^⋅min^–1^ per year).

**TABLE 2:** 20mSRT performance outcomes including last completed stage, estimatedV̇O_2peak_ (mL⋅kg^–1^⋅min^–1^), and peak heart rate (HR) at the end stage, stratified by age, sex and generation. Peak HR included for the 2003 and 2013 generations only.

**Age (y)**	**All**			**Boys**			**Girls**		
**End Stage (#)**	**1993**	**2003**	**2013**	**1993**	**2003**	**2013**	**1993**	**2003**	**2013**
7	2.0 ± 0.8	1.8 ± 0.9	3.3 ± 1.2	2.2 ± 0.9	1.9 ± 1.0	3.4 ± 1.3	1.9 ± 0.8	1.7 ± 0.8	3.2 ± 1.1
8	2.8 ± 1.3	2.3 ± 1.2	3.9 ± 1.5	3.1 ± 1.4	2.4 ± 1.3	4.2 ± 1.7	2.6 ± 1.2	2.2 ± 1.1	3.7 ± 1.4
9	3.2 ± 1.6	2.9 ± 1.4	4.6 ± 1.7	3.4 ± 1.6	3.1 ± 1.6	4.9 ± 1.9	3.1 ± 1.5	2.6 ± 1.2	4.3 ± 1.4
10	4.0 ± 1.7	3.7 ± 1.7	4.9 ± 1.8	4.4 ± 1.9	4.2 ± 1.8	5.4 ± 2.0	3.7 ± 1.5	3.2 ± 1.5	4.5 ± 1.6
11	4.7 ± 1.9	4.3 ± 1.9	5.4 ± 2.0	5.2 ± 2.0	5.0 ± 1.9	5.7 ± 2.2	4.2 ± 1.7	3.5 ± 1.4	5.1 ± 1.7
12	5.3 ± 1.8	4.5 ± 1.8	6.1 ± 2.1	5.8 ± 2.0	5.1 ± 1.9	6.5 ± 2.2	4.8 ± 1.6	3.8 ± 1.5	5.6 ± 1.8
13	5.6 ± 2.0	4.9 ± 2.0	6.5 ± 2.1	6.5 ± 2.0	5.5 ± 2.1	6.9 ± 2.4	4.7 ± 1.6	4.2 ± 1.6	6.0 ± 1.8
14	5.9 ± 2.2	5.3 ± 1.9	6.9 ± 2.3	6.9 ± 2.0	6.1 ± 2.0	7.8 ± 2.3	4.7 ± 1.7	4.4 ± 1.5	5.9 ± 1.8
Mean total	4.2 ± 2.2^b,c^	3.7 ± 2.0^a,c^	5.1 ± 2.2^a,b^	4.7 ± 2.4^b,c^	4.2 ± 2.2^a,c^	5.5 ± 2.4^a,b^	3.7 ± 1.8^b,c^	3.2 ± 1.6^a,c^	4.6 ± 1.8^a,b^
V̇O_2peak_(mL⋅kg^–1^⋅min^–1^)									
7	47.1 ± 1.9	46.6 ± 2.0	49.9 ± 2.6	47.5 ± 2.0	46.8 ± 2.2	50.2 ± 2.9	46.8 ± 1.7	46.4 ± 1.8	49.7 ± 2.3
8	47.0 ± 2.9	45.9 ± 2.6	49.5 ± 3.4	47.6 ± 3.2	46.2 ± 2.7	50.1 ± 3.6	46.6 ± 2.6	45.6 ± 2.5	49.0 ± 3.0
9	46.2 ± 3.7	45.4 ± 3.3	49.4 ± 3.9	46.5 ± 3.8	46.0 ± 3.7	50.0 ± 4.4	45.9 ± 3.6	44.9 ± 2.6	48.7 ± 3.1
10	46.3 ± 4.0	45.5 ± 4.0	48.5 ± 4.3	47.2 ± 4.4	46.9 ± 4.2	49.5 ± 4.7	45.5 ± 3.5	44.4 ± 3.4	47.6 ± 3.8
11	46.3 ± 4.7	45.2 ± 4.5	47.9 ± 4.8	47.5 ± 4.8	47.0 ± 4.7	48.6 ± 5.4	45.2 ± 4.2	43.3 ± 3.5	47.2 ± 4.2
12	46.1 ± 4.6	44.1 ± 4.6	48.2 ± 5.2	47.4 ± 5.0	45.6 ± 4.8	49.3 ± 5.6	44.8 ± 3.9	42.4 ± 3.7	47.0 ± 4.4
13	45.3 ± 5.3	43.5 ± 5.2	47.5 ± 5.6	47.7 ± 5.3	45.1 ± 5.4	48.7 ± 6.1	42.9 ± 4.2	41.6 ± 4.2	46.4 ± 4.7
14	44.5 ± 5.8	42.8 ± 5.2	47.3 ± 6.2	47.2 ± 5.5	45.0 ± 5.2	49.5 ± 6.2	41.5 ± 4.5	40.4 ± 4.0	44.5 ± 5.0
Mean total	46.1 ± 4.4^b,c^	44.9 ± 4.3^a,c^	48.6 ± 4.6^a,b^	47.3 ± 4.4^b,c^	46.1 ± 4.3^a,c^	49.6 ± 4.9^a,b^	44.9 ± 4.0^b,c^	43.7 ± 3.8^a,c^	47.7 ± 4.1^a,b^
Slope (mL⋅kg^–1^⋅min^–1^)/year	−0.38	−0.54	−0.37	−0.04	−0.26	−0.09	−0.76	−0.86	−0.75
Peak Heart Rate (b⋅min^–1^)									
7	–	193 ± 14	199 ± 12	–	193 ± 11	199 ± 14	–	193 ± 17	199 ± 11
8	–	193 ± 15	199 ± 11	–	191 ± 14	199 ± 11	–	195 ± 15	199 ± 11
9	–	197 ± 11	200 ± 14	–	196 ± 11	200 ± 13	–	198 ± 10	201 ± 14
10	–	198 ± 11	201 ± 10	–	198 ± 12	201 ± 10	–	199 ± 9	202 ± 10
11	–	199 ± 11	201 ± 11	–	199 ± 10	200 ± 12	–	199 ± 12	202 ± 10
12	–	199 ± 10	202 ± 10	–	199 ± 9	201 ± 11	–	199 ± 10	204 ± 9
13	–	200 ± 10	202 ± 10	–	199 ± 9	200 ± 12	–	201 ± 11	203 ± 9
14	–	198 ± 10	201 ± 11	–	198 ± 10	201 ± 10	–	199 ± 10	201 ± 11
Mean	–	197 ± 12	201 ± 11^b^	–	197 ± 11	200 ± 12^b^	–	198 ± 12^d^	201 ± 11^b,d^

*“Slope” refers to the rate of reduction in V̇O_2peak_ per year from age 7 to 14 for that sex, within that generation.*

*(^*a*^) Significantly different from 1993 generation, within-sex,*

*(^*b*^) Significantly different from 2003 generation, within-sex,*

*(^*c*^) Significantly different from 2013 generation, within-sex,*

*(^*d*^) Significantly different from boys of same generation.*

**FIGURE 1:**
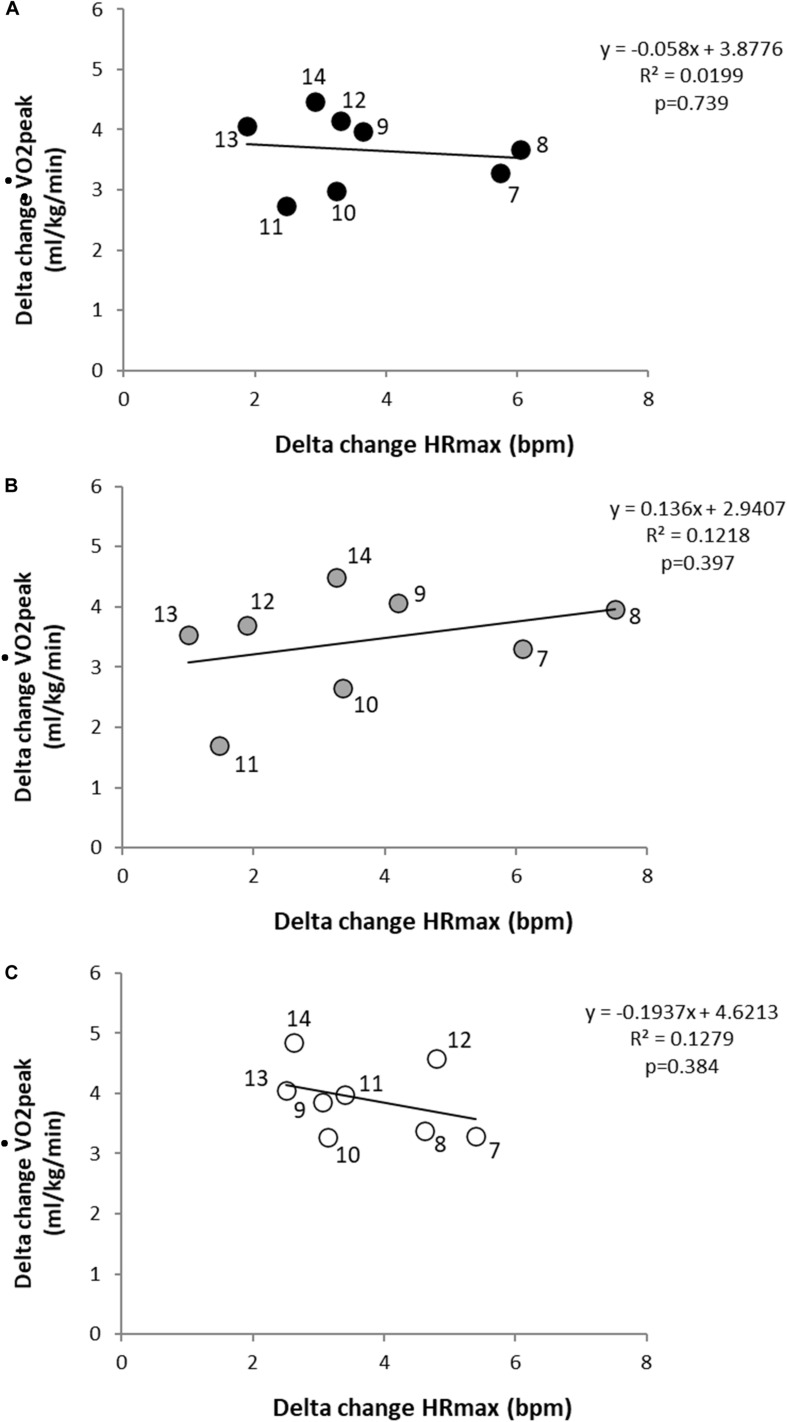
Delta change scores between the 2003 and 2013 generations in aerobic fitness and maximum heart rate for **(A)** all **(B)** boys, and **(C)** girls. Scatterplots depict mean change for a given age group (age labeled beside each dot).

Peak heart rate (HR_peak_) was marginally lower (by ∼1 beat) in boys than girls (95% CI: 197.9−198.7 versus 199.2−200.0 b⋅min^–1^, *p* < 0.001); differences in HR_peak_ were consistently greater between-generation by 3.1−4.2 b⋅min^–1^ (*p* < 0.001, Table 2). For every Δ HR_peak_ of ∼2-6 b⋅min^–1^ there was a corresponding increase ΔV̇O_2peak_ of 1.5−5.0 mL⋅kg^–1^⋅min^–1^ (Figure 1).

### Other Supporting Measures

Children at age 7 spend ∼36 fewer minutes watching screens than 10-year-olds (95% CI: −52.9−−20.6 min, *p* < 0.001, Table 1). Children reported sleeping less each year, such that 11-year-olds slept nearly 1 full hour (∼0.89 h) longer than 14-year-olds (95% CI: 0.5−1.2 h, *p* < 0.001). MVPA was lower in girls compared to boys for ages 12, 13, and 14 (each *p* < 0.001; 95% CI: 8.7−16.1 (12 year), 8.7−16.2 (13 year) and 7.7−17.2 min (14 year), respectively).

### Estimation of Cardiovascular Health Risk

**FIGURE 2:**
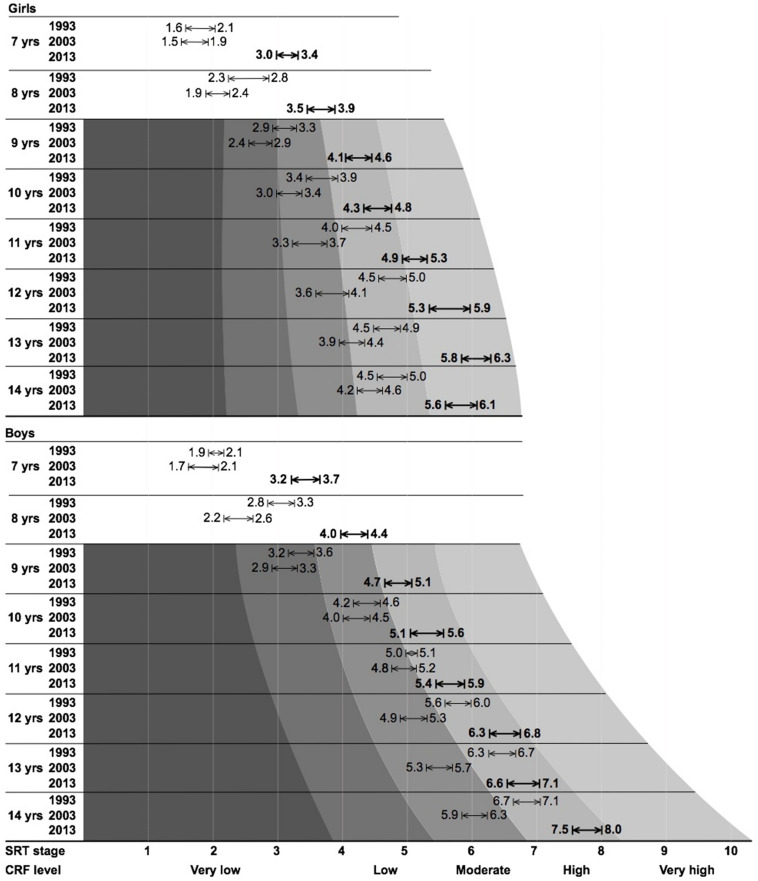
Final stage completed for each generation of Slovenian schoolchildren, contrasted with worldwide centile normative data (where available). Values listed correspond to the lower- and upper-bound 95% confidence intervals.

**FIGURE 3:**
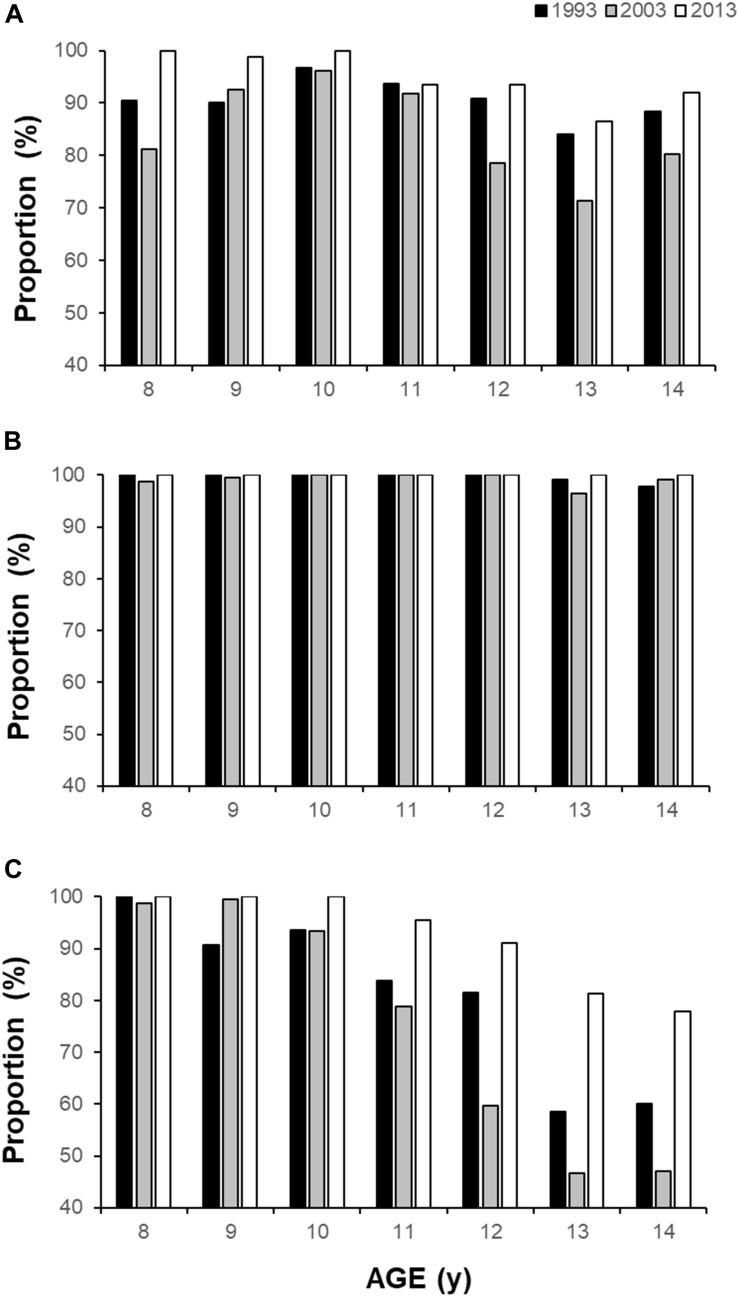
Proportion of children who meet (or exceed) the CRF cut-off value for reduced health risk for **(A)** boys (41.8 ml⋅kg^–1^⋅min^–1^), **(B)** girls (34.6 ml⋅kg^–1^⋅min^–1^), and **(C)** proportion of girls who meet or exceed the boys’ cut-off standard, stratified by age and generation.

The 95% CI of final stage completed for each generation was contrasted with worldwide centile normative data. Centiles varied widely between generation and sex; for example, for a given age and generation of boys, the 95% CI fell within “very low” (0–20%) up to “high” (60–80%), whereas girls fared somewhat better, ranging between “low” (20–40%) and “very high” (80–100%, Figure 2). The proportion of children who met (or exceeded) CRF cut-off values for reduced cardiovascular health risk (boys = 41.8 mL⋅kg^–1^⋅min^–1^, girls = 34.6 mL⋅kg^–1^⋅min^–1^) were always above 50% for any given population (Figure 3).

## Discussion

This investigation found consistent declines in CRF across all ages, regardless of sex, from 1993 to 2003, before rebounding to their highest levels in 2013. Children were able to attain higher HR_peak_ in 2013 compared to 2003, suggesting this cohort was able to achieve, maintain and/or tolerate higher levels of cardiovascular strain at the 20mSRT endpoint. The rate of decrease in girls’ CRF was worse in 2003 compared to other generations. Slovenian schoolchildren achieve minimum CRF cut-off point to avoid cardiovascular health risks, ranging from 75 to 100% for any given generation.

### Secular Trends in Slovenia

Cardiorespiratory fitness decreased by ∼2.8% from 1993 to 2003 for both boys and girls, as estimated by the original Léger equation. There is continued debate on the best, or rather, most accurate model to assess 20mSRT as a predictor of CRF (Welsman and Armstrong, 2019). Knowing these limitations, it remains that initial decreases in CRF were reversed from 2003 to 2013 (∼8.2%), across all ages, in both sexes and in-line with overall secular trends of physical fitness in Slovenia during that time (Potočnik et al., 2020). Indeed, CRF increased ∼5.4% across the 20-year (1993–2013) period of study. The rate of reduction in CRF from age 7 to 14 (i.e., the slope of the response) was lowest in 2013 (i.e., more favorable). Since participants must move their entire body mass against the forces of gravity, we report 20mSRT relative to total body mass of the individual. Emerging research is considering updating the original Léger equation in favor of a curvilinear allometric model (Nevill et al., 2020). To what extent new models may impact calculated risk assessments, centile normative values, or test interpretation, remains unclear. What is clear from this reporting is that, at a population level, the CRF of Slovenian schoolchildren remain relatively high, possibly since Slovenia maintains some of the highest levels of child PA in the world (Sember et al., 2018).

### Possible Drivers of Generational Change in CRF

Between 1993 and 2003, CRF in Slovenian schoolchildren was systematically worsening, reflecting global trends for this metric (Tomkinson et al., 2003), likely due to several factors. For one, changes in Slovenia’s political system (especially since 1991) affected family economics such that economically weaker ones could not enroll children in expensive sporting programmes (Jurak et al., 2002a). Additionally, the sheltering praxis of parents promoted restricting child access to public spaces (e.g., playing on playgrounds, walking alone), individualization, and sedentary lifestyle each became commonplace amongst Slovenian children (Starc and Kovač, 2007). Moreover, it was found that physical education (PE) teachers were omitting some physically intensive content in their classes (Bučar Pajek et al., 2010), and these PE lessons (especially at lower ages) were often taught by general practitioners less effective in class organization (Jurak et al., 2006). With a mean sleep time of 9.4 ± 1.2 h, children are generally achieving enough cumulative sleep to meet international standards for that metric (Paruthi et al., 2016) and was not considered a factor in the generational changes observed in CRF.

There was a combination of societal changes in the 2000’s, including several systematic interventions at the national level, that did contribute to more favorable trends in PA, which is reflected in the improved CRF scores from 2003 to 2013. Namely, this period was marked by the implementation of new PE curricula which provided almost all schoolchildren (aged 6–14 year) with 135 min of PE per week, delivered by specialist PE teachers from age 11 onwards, and taught separately by gender (Jurak et al., 2020b). Furthermore, because increasing trends in childhood obesity and declining fitness across several metrics were observed within the national child physical fitness surveillance system (SLOfit) (Jurak et al., 2020a), a national PA intervention program entitled “Healthy Lifestyle” (HLS) was implemented from 2010/11, which provided two additional PE hours per week, delivered by specialist physical educators for students 6–14 years old (Jurak et al., 2020b). HLS was considered part of a school’s extracurricular health-oriented PA program and included more than 30% of the entire elementary school population during its tenure. HLS was a coordinated effort by the national government to inject greater PA volume into the school system, ultimately exposing children to up to 51 min of daily PA (Sember et al., 2016) they would otherwise not have received.

### Normative Reference Standards for Estimating Health Risk as a Pedagogical Tool for Physical Literacy

Health practitioners, clinicians and teachers propose applying international standards of CRF more readily in clinical and school settings (Lang et al., 2019). Normative CRF data, similar to international BMI growth curves developed for children and youth (Ruiz et al., 2016), can be a helpful tool to identify children who need to increase PA, improve overall health, and reduce their risk of future disease-state outcomes. For practical use, CRF standards-based feedback should provide experts with a comprehensive way to increase the physical literacy of students and their parents (Whitehead, 2001). Based on the results of this study, the question arises as to whether existing CRF normative-referenced standards for estimating health risk (Ruiz et al., 2016) fulfill this role. In particular, almost all Slovenian schoolchildren herein meet international guidelines for healthy CRF. However, when examining the results in detail, Slovenia can have 9-year-old boys in the “very low” to “low” centile range, but 80–90% of them are achieving the cut-off point for low cardiovascular risk. This discrepancy is exacerbated in girls such that, for any generation, nearly 100% of all girls met the published minimum health risk standards. Such discrepancies are also common in populations that are less fit (Tomkinson et al., 2018). Indeed, having a minimum standard of 9.0 km/h (especially in the younger age groups) is a somewhat controversial, since the 20mSRT test starts at a speed of 8.5 km/h, children need only complete one stage to achieve a “low-risk” health status. The authors posit that perhaps we are entering a new phase of 20mSRT fitness testing; in general, it has proven that when measured correctly the test is reliable and valid, and research groups are continuing to hone the predictive model validity of the measure (Nevill et al., 2020), but now we must harmonize these research findings to a more meaningful pedagogical and translational public health tool.

### Strengths, Weaknesses and Study Considerations

The 20mSRT was measured at the same locations for each iteration of data collection. Despite this, there are many factors that can affect test performance, including: environmental conditions, clothing, field surfaces, footwear, motivation, pre-test instructions, and diurnal variation. The current study makes every effort to standardize these factors. Testing took place in the fall semester, indoors, in a gym, with children wearing light, athletic clothing, barefoot, having standard pre-test instructions, and always in the morning. Standardized fitness testing is a common and consistent tool measured annually in Slovenia [SLOfit (Jurak et al., 2020a)], so although there are likely motivational factors which vary across individual schoolchildren, they are nonetheless accustomed to systematic fitness testing in schools. Peak heart rate was available only during the 2003 and 2013 generation. A 20mSRT data were recorded as last stage completed, not consistently in total laps. We therefore calculated speed at last stage and estimated V̇O_2peak_but were not confident in reporting total laps for all generations herein. Involvement in this study was voluntary and based on informed parental consent. Since the physical fitness test could represent a risk to children with certain chronic diseases, only healthy children without injuries were included in the study. It is not possible to assess the reasons why parents might not have given positive consent for their children which would have resulted in rejection of their child’s participation. Among those (few) parents who did opt out, it is possible the reason could have been due to certain chronic or acute health conditions, but since there is no personal data to consider, we cannot speculate on any possible difference(s) between children included in this study, and those who did not participate.

## Conclusion

Negative trends in CRF from Slovenian schoolchildren were reversed by 2013, indicating that Slovenia should continue implementing the progressive national physical fitness strategies introduced between sampling years (e.g., 2003–2013). Additionally, due to the universal nature of Slovenian schoolchildren achieving the cut-off points for reduced health risk, it is proposed that more specific benchmark criteria are developed so the results can be more accurately utilized in clinical and pedagogical settings. Cut-off values for healthy zones in younger ages should be revised in-line with more modern methods of calculating CRF. Further investigations of CRF secular trends may include accounting for generation changes in mass, maturation, body composition and other environmental factors known to significantly affect performance outcomes.

## Data Availability Statement

The raw data supporting the conclusions of this article will be made available by the authors, without undue reservation.

## Ethics Statement

The studies involving human participants were reviewed and approved by the Slovenia National Medical Ethics Committee (ID: 138/05/13). Written informed consent to participate in this study was provided by the participants’ legal guardian/next of kin.

## Author Contributions

SM: conceptualization, formal analysis, visualization, writing – original draft, writing, review, and editing. VS: investigation, data curation, formal analysis, and writing – original draft. BL: data curation, formal analysis, writing, review, and editing. MK: investigation, resources, writing, review, editing, and project administration. GJ and GS: conceptualization, investigation, resources, writing, review, editing, and project administration. All authors contributed to the article and approved the submitted version.

## Conflict of Interest

The authors declare that the research was conducted in the absence of any commercial or financial relationships that could be construed as a potential conflict of interest.
